# RETRACTION: The efficacy of pomegranate peel extract on *Eimeria* shedding and growth indices in experimental coccidiosis in broiler chickens

**DOI:** 10.1002/vms3.1564

**Published:** 2024-07-25

P. Khorrami, M. Gholami‐Ahangaran, E. Moghtadaei‐Khorasgani, “The efficacy of pomegranate peel extract on *Eimeria* shedding and growth indices in experimental coccidiosis in broiler chickens,” *Veterinary Medicine and Science* 8, no.2 (2022): 635–641, https://doi.org/10.1002/vms3.714.

The above article, published online on 04 January 2022 in Wiley Online Library (wileyonlinelibrary.com) has been retracted by agreement between the journal's Editor‐in‐Chief, Gayle Hallowell, and John Wiley & Sons Ltd. An investigation by the publisher found this article to have experienced compromised peer review. Based on the investigation's findings, the Editor‐in‐Chief no longer has confidence in the reported results and conclusions drawn. Therefore, a decision has been made to retract this article. The authors have been informed of the decision to retract but do not agree with this decision.

